# Revealing hidden depression in older people: a qualitative study within a randomised controlled trial

**DOI:** 10.1186/s12875-015-0362-2

**Published:** 2015-10-19

**Authors:** Karen Overend, Katharine Bosanquet, Della Bailey, Deborah Foster, Samantha Gascoyne, Helen Lewis, Sarah Nutbrown, Rebecca Woodhouse, Simon Gilbody, Carolyn Chew-Graham

**Affiliations:** University of York, Heslington, York, YO10 5DD UK; Research Institute, Primary Care and Health Sciences, NIHR CLAHRC West Midlands, Keele, Staffordshire ST5 5BG UK

**Keywords:** Depression, Older people, Collaborative care, Primary care, Qualitative method

## Abstract

**Background:**

The prevalence of depressive symptoms in older people may be as high as 20 %. Depression in older people is associated with loss, loneliness and physical co-morbidities; it is known to be under-diagnosed and under-treated. Older people may find it difficult to speak to their GPs about low mood, and GPs may avoid identifying depression due to limited consultation time and referral options for older patients.

**Methods:**

A nested qualitative study in a randomised controlled trial for older people with moderate to severe depression: the CASPER Plus Trial (Collaborative Care for Screen Positive Elders). We interviewed GPs, case managers (CM) and patient participants to explore perspectives and experiences of delivering and receiving a psychosocial intervention, developed specifically for older adults in primary care, within a collaborative care framework. Transcripts were analysed thematically using principles of constant comparison.

**Results:**

Thirty three interviews were conducted and, across the three data-sets, four main themes were identified: revealing hidden depression, reducing the ‘blind spots’, opportunity to talk outside the primary care consultation and ‘moving on’ from depression.

**Conclusions:**

Depression in older people is commonly hidden, and may coexist with physical conditions that are prioritised by both patients and GPs. Being invited to participate in a trial about depression may allow older people to disclose their feelings, name the problem, and seek help. Offering older people an opportunity to talk outside the primary care consultation is valued by patients and GPs. A psychosocial intervention delivered by a case manager in the primary care setting may fill the gap in the care of older people with depression.

**Trial registration:**

Current Controlled Trials ISRCTN45842879.

## Background

### Introduction

Estimates of the prevalence of depression in older people vary but may be as high as 20 % [1, 2] Poor mental health is often co-morbid with long term, chronic physical illness [3] such as diabetes, coronary heart disease, stroke and Parkinson’s disease, all of which are more common in later life. Depression reduces quality of life and increases the risk of suicide [4]. Depression also increases use of health and social care, including use of unscheduled care [5].

Loneliness and depression are strongly associated in older people [6] and loneliness is an independent risk factor for depression [7, 8]. Barriers to identifying and managing depression in an older population are complex [9–11] Older people with long term conditions (LTCs) may view their chronic illness as a justifiable cause of low mood, and may hold negative views about available treatment options [10, 11]. Older people may be reluctant to define their distress as a mental health problem because of the perceived stigma associated with the label ‘depression’, and thus be reluctant to consult, not considering their emotional health as a subject worthy of their GP’s attention. Burroughs et al. [9] reported that diagnosis and treatment can emphasise the biomedical model with less attention paid to social and contextual issues [9]. Coventry et al. [12] reported that depression was often ‘normalised’ among those with chronic physical health problems. A one-year follow-up study by Licht-Strunk et al. [13] showed, compared to depressed older patients identified by their GPs, prognosis of those with undetected depression is poor.

Older people may experience difficulty in accessing mental health care, which may be due to a lack of capacity within the healthcare system with adults over 65 being less likely than younger people to be referred to IAPT services [14]. Thus, older people are an important ‘under-served’ group, increasingly affected by economic deprivation, social isolation and loneliness [15]. With the growing ageing population there is an increasing need for an intervention specifically developed for older people with depression.

Evidence shows that Behavioural Activation (BA) is an effective treatment for depression [16], particularly in older people [17, 18]. BA forms the cornerstone of a number of recent trials where the intervention was delivered by psychological well-being practitioners (PWPs) within a collaborative care model [19]. This framework, defined by Gunn [20] incorporates: a multi-professional approach to patient care, a structured management plan, scheduled patient follow-ups and enhanced communication between professionals (See Table 1).

**Table 1:** The collaborative care framework (Gunn et al. [20])

Multi-professional approach to patient	Care provided by a case manager working with the patient’s GP and under supervision from a specialist mental health clinician
Structured management plan	Brief psychological therapy e.g. BA (plus/- medication support)
Scheduled patient follow-ups	Proactive care
Enhanced inter-professional communication	Patient-specific written feedback to GPs via letter, email and personal contact

A reasonable evidence base exists for the role of collaborative care (CC) in improving outcomes for people with depression. Thus, a large US study [21] shows evidence of its effectiveness as a treatment for depression in adults over 60, and in the UK the CADET study [19, 22] reported persistent positive effects of collaborative care in improving depression among a general adult population, up to 12 months after initiation of the intervention. The COINCIDE study of collaborative care for adults with co-morbid depression and diabetes or cardiovascular disease also showed positive results [23]. The CASPER Plus trial was developed specifically to evaluate the clinical and cost effectiveness of collaborative care for older people with moderate to severe depression in a UK setting [24].

Simpson et al. [25] reported on the experiences of depressed patients receiving collaborative care in the UK, suggesting case managers were able to reduce the sense of stigma at being diagnosed with a mental health problem and resolve misconceptions around antidepressant medication prescribed by the GPs. However, Knowles et al. [26] reported that established divisions between physical and mental health continue to pose barriers for the management of people with long term conditions and depression; and identified a need among such patients for a separate space to discuss psychological concerns [27].

Little work has been done to explore the perspectives of older adults with depression being offered and receiving treatment for depression within a CC framework. Through semi-structured interviews, our aim was to investigate both patient and professional views in order to gain multiple perspectives on the understanding of depression and depression management in older people.

## Methods

### Ethical approval

Nested in the CASPER Plus study [24], a pragmatic randomised controlled trial (RCT), we conducted a qualitative evaluation using semi-structured interviews across three sites in the north of England to explore acceptability, views and experiences of GPs, trial participants (patients) and case managers. Ethical approval for the RCT and this qualitative study was gained by Leeds East Research Ethics Committee, Yorkshire & Humber (reference 10/H1306/61).

### Recruitment and sampling

We aimed to interview a purposive sample of GPs, patient participants and those who declined to participate or withdrew from the intervention, and case managers. Our approach was to sample patients and GPs from recruiting practices in both urban and rural areas in the north of England, in order to gather data from areas of differing deprivation indices and achieve a spread in gender, age and socioeconomic status.

### Interview schedule and selection process

Participants from three groups were invited to take part in an interview: GPs working within CASPER Plus trial practices, patients who consented to participate and were randomised to CC and case managers who delivered the intervention.

Invitation letters, with an information leaflet describing the interview process and a consent form with a pre-paid envelope to return to the research team, were sent to patient participants by post; GPs and CMs were sent invitation letters with an information leaflet and a consent form by email. Before interviews commenced, written informed consent was obtained from all participants.

Initially, all GPs and patient participants who had completed their part in the trial were invited to be interviewed. In the trial [24] GP practices were recruited sequentially, each practice then inviting eligible patients over 65 years to participate in the trial. Once five or more patients from each practice had completed the intervention, the lead GP was invited to be interviewed in the qualitative study.

All patient participants were invited to be interviewed after they had completed the intervention. Non-responders in both groups were followed up by telephone. We aimed to interview two groups of patient participants: those who completed treatment and those who withdrew from treatment.

At the start of our study, following the order of GP practice recruitment, all participants invited to take part were from the central site: urban and rural practices in York, Harrogate, Hull and surrounding areas. As these are areas of relatively low to moderate deprivation, we then used a selection process to ensure that participants from areas of higher deprivation were invited to be interviewed.

Of the 18 GPs invited to take part, 12 consented to be interviewed. All 12 case managers who took part in the trial were invited to be interviewed, all of whom had received training and delivered the intervention to at least three patient participants.

### Data collection

Interviews were carried out by KO, KB and SN at a place convenient to the participant: either at the GP practice (for GPs), at the home of the patient participant or, for CMs, in the researcher’s office. Interviews were conducted between May 2013 and November 2014. All interviews were digitally recorded (with participants’ signed consent), transcribed verbatim and anonymised. The topic guides were designed with reference to the literature, approved by the research team, and developed iteratively in light of the emerging themes.

### Analysis

The transcriptions formed the data which were then analysed iteratively, allowing for modification of the topic guides as analysis progressed. Analysis was undertaken by individual researchers KO, CCG and KB (of different professional backgrounds: health sciences research, academic primary care, academic health research) using thematic analysis and principles of constant comparison [28] and Framework [29] methods. Data analysis involved a process of organising the data, descriptive coding, interpretive coding, writing and theorising. Deviant cases were actively sought throughout the analysis and emerging ideas and themes modified in response. Following analysis by individual researchers, themes were agreed during face-to-face discussion with the full research team.

## Results

Twelve GPs, 13 patient participants (of whom 12 had completed the intervention, one withdrew before starting therapy) and 8 case managers were interviewed (see Tables 2, 3 and 4).

**Table 2:** Demographics of patient participants

	Gender	Age range	IMD^a^	Face to face/telephone	From urban or rural GP practice
PT1	F	75–80	1	Face to face	Urban
PT2	M	75–80	9	Face to face	Urban
PT3	M	65–70	5	Face to face	Rural
PT4	M	81–85	8	Face to face	Rural
PT5	M	65–70	2	Face to face	Urban
PT6	F	65–70	10	Face to face	Rural
PT7	F	65–70	10	Face to face	Rural
PT8	F	65–70	10	Face to face	Urban
PT9	M	65–70	2	Face to face	Urban
PT10	F	65–70	8	Telephone	Urban
PT11	F	75–80	9	Face to face	Urban
PT12	F	65–70	9	Telephone	Urban
PT1W	M	65–70	6	Face to face	Rural

*W* withdrawn

^a^Index of Multiple Deprivation. Lower numbers indicate lower socioeconomic status

**Table 3:** Demographics of GPs interviewed

ID	Gender	Practice size	IMD^a^	Urban or rural GP practice
GP1	M	14,886	5	Urban
GP2	M	10,150	6	Urban
GP3	M	19,879	10	Rural
GP4	F	18,083	8	Rural
GP5	M	24,353	5	Urban
GP6	M	15,915	4	Urban
GP7	M	6961	6	Urban
GP8	F	13,000	3	Urban
GP9	F	18,083	8	Rural
GP10	F	11,893	6	Rural
GP11	M	7183	10	Rural
GP12	M	15,432	5	Rural

^a^Index of Multiple Deprivation. Lower numbers indicate lower socioeconomic status

We were less successful in recruiting GPs and patients from more deprived areas

**Table 4:** Demographics of CMs interviewed

ID	Gender	Years of experience^b^	Interview type (face to face & telephone/telephone only)
CM1	F	8	Face to face
CM2	F	9	Face to face
CM3	F	4	Face to face
CM4	F	4	Face to face
CM5	F	4	Telephone
CM6	F	3	Telephone
CM7	F	3	Telephone
CM8	F	5	Face to face

^b^experience in years of delivering a low-intensity psychological intervention

Only one patient was interviewed who had withdrawn from the study. Most trial participants who withdrew did so at the outset, either declining to receive the intervention or withdrawing after only one session. It was difficult to recruit such older people to participate in an interview. As an ethical consideration, our protocol stated that after inviting withdrawn participants by letter to take part in an interview, we would not follow up those who did not respond.

Eight case managers consented to be interviewed. The reasons for the four declining are unclear, but all worked in the site which was last to join the study; they saw fewer patients than other case managers and were on short-term, part-time contracts.

We report the main themes identified in the data: Revealing hidden depression, reducing the ‘blind spots’, opportunity to talk outside the primary care consultation and ‘moving on’ from depression.

Data is presented to support analysis and labelled by identifier and number: CM = case manager; PT = patient; PTW = withdrawn patient; GP = general practitioner.

### Revealing hidden depression

For most of the older people we interviewed, being invited to participate in the CASPER Plus study seemed to raise awareness of low mood:

*“It crept up on me really, how I felt. I think it had been coming on for a long time and I didn’t realise how bad I’d got until I filled that form in and I just ticked the boxes and posted it.”* PT6

Several GPs described how taking part in the CASPER plus trial helped to raise awareness of depression in their older population. One GP said:

*“I think it has probably alerted us to one or two of the… more needy patients who perhaps were not coming to us for help… people have been brought into the system that… had sort of dropped out from seeing the GP.”* GP3

Some case managers (CMs) described how some patient participants admitted they had not spoken to others, including their GPs, about how low they felt.

*“One gentleman that I saw, he said the most useful thing had been the diagnostics, as risk was identified, and so we wrote to the GP about that. And it was… the risk was still there when I saw him for the first time so I put that in a letter as well and he said that had kind of opened the door. He would have never gone and spoken to his GP about it.”* CM2

*“they (the patient) wouldn’t do anything and they wouldn’t commit suicide but they feel ashamed I guess of having some thoughts (that they’d be better off dead)… and those are the sorts of things they don’t always like us to share with the GP because it’s back to that stigma isn’t it?”* CM1

While some patient participants did not use labels such as ‘depression’ or ‘low mood’, those who did so suggest that *other* older people may fail to recognise or admit their feelings because of the perceived stigma of doing so:

*“…people don’t talk about it do they, they think it’s a weakness don’t they? But it is something that you can’t help when you are in it, you know as I say you don’t realise you are going in it and as much as you try you know sometimes you can’t get out it, it gets deeper you know.”* PT6

A few patient participants commented on the ‘invisibility of depression’:

*“…you know if I broke an arm I’d get a sling wouldn’t I, you know it’s fairly obvious, but I suppose with any mental illness you can’t see it, you don’t know.”* PTW1

Several GPs reported an awareness of the stigma associated with depression, especially in this age group, which might impact on whether it is raised within a consultation:

*“It’s sort of an age group where they’re not as open about depression as maybe younger people are, there’s a bit of a stigma attached to it still.” GP8*

A few GPs described how they *normalised* depression in older people; one admitted possibly colluding with the patient in ignoring cues within the primary care consultation:

*“You’re sort of aware there are people who have depressive episodes that aren’t possibly addressed, they may themselves not really recognise it, and they just think it’s part of, you know, getting older.”* GP3

*“You’d like to think that primary care is fairly aware of it (depression) anyway. But maybe the temptation is to let sleeping dogs lie, I don’t know. So you know, if you diagnose someone with depression you’ve got to do something about it haven’t you?”* GP6

Some GPs described a tension between a desire to consider the ‘whole’ patient and, due to limited time and treatment options, a tendency to prescribe antidepressants to older people who they did recognise as being depressed.

*“We often go down a medication route because, well it does help them, and it’s very difficult to get other services. And the psychiatry for the elderly tends to be more focused on dementia.” GP8*

Several GPs recognised that depression in older people often occurs alongside complex physical conditions or social problems, including loneliness. Some of these GPs disclosed a reluctance to identify the condition, partly due to the absence of a psychological treatment pathway for depression in the over-65 s and a tendency to prioritise physical symptoms over emotional health.

*“I suppose in a busy clinic we probably don’t have time to sort of delve into depression along with the sort of twelve and a half minutes of consulting on chronic diseases that’s squeezed into ten minutes, so depression would take another five or six, so… we’ll probably skip over that unless they bring it to us.”* PT12

Being invited to participate in the CASPER Plus trial provided an opportunity for some people to talk about depression, enabling them to recognise and seek help for low mood.

### Reducing the ‘blind spots’

Several case managers and three GPs described how two practitioners working with a patient helped to reduce the ‘blind spots’, as each professional offered a different perspective.

“*so you’ve got the benefit of somebody who’s looking at a person, never having met them before who can see certain things, versus somebody who has known somebody for some time and can see certain things but, those two people, will have, probably have, blind spots… because one person doesn’t know that person very well and the other has maybe, over the years, has just sort of formed a fixed idea about somebody. Collaborative working, not only will it progress the patient forward but it will also… reduce blind spots, I think, in their care*.” GP1

One GP saw the CM as helping to ‘patch up’ the gaps in the patient’s support network.

“*I think a lot of the difficulty… is their support networks have become a bit more fragmented…. especially those that are bereaved, or have families spread around the country or spread around the world… so I can see that maybe we can patch that fragmentation up a little bit… it’s not the same as having your relatives but having some kind of support, I can see that as a benefit*.” GP3

The CMs viewed their role as a facilitator, or ‘go-between’, able to convey information to the GP which the patient may be reluctant to disclose directly.

“*Sometimes, if people can’t talk to their GP or don’t understand that maybe they had a problem like depression, and don’t know how to approach a GP because of stigma and things like that then I’ve been that facilitator, I’ve helped them with that process*.” CM1

For example, one CM reported advocating on behalf of a patient who was having problems with pain:

*“…she was using cannabis to manage the pain and she felt there was nothing else the doctors could do, so I spoke to her GP and they said she could get a referral to the pain clinic… She [the patient] had given up all hope, but she was happy for me to pester them a little bit.”* CM3

GPs and CMs offered different perspectives on patients’ health needs which was seen to reduce ‘blind spots’ in depression care.

### Opportunity to talk outside the primary care consultation

Offering an opportunity to talk outside the GP consulting room was valued by the majority of patients:

*“The most startling thing about the experience was all my life I’ve never had anybody to talk to, there’re things I wouldn’t even discuss with my wife and to have an outsider person that didn’t really know me who was impartial… that helped me a great deal, just by having someone to discuss things with.”* PT5

*“…having someone to talk to… about things in my life that I would talk to say the family about or friends unless they were extremely close friends, it gave me someone objective to talk to you know, that was removed from my situation.”* PT2

Some patients suggested that GPs were not always receptive to discussing problems with mood:

“*You know and the GPs, well they don’t, they don’t seem to be interested I don’t think. Oh, it’s depression, take a pill, go away.”* PT12

*“I just have a bit of a problem with doctors because I just don’t think they do the job that they maybe should be doing, it’s a two minute interview or whatever, they don’t really know your records, they don’t know the history, they don’t tie things up.”* PTW1

In contrast, most patients described the CM as providing empathic support, being able to offer more time than the GP and knowing where to signpost patients to voluntary organisations:

*“…she did everything she possibly could… I mean she went the extra mile. She spoke to the people at Parkinson’s – Parkinson’s UK – to see if there was a network somewhere, an advice centre, and things I didn’t know she found out for me.”* PT7

Patient participants spoke about the benefit of having someone to talk to in confidence, outside the primary care consultation; someone who was said to listen without judging, allowing them to talk openly about feelings and personal issues:

*“I thought it was very good. And I think the fact that people were bothered, to see how the older people felt…. I think that was good. You didn’t feel like you just got a script thrown at you and you were waiting for God sort of thing…it was the fact that someone was interested in how you felt.” PT1*

Giving patients an opportunity to talk outside of the clinical setting of the primary care consultation room appears to be valued by most of the older people we interviewed, and by their GPs.

### ‘Moving on’ from depression

Some patients reported how the CM encouraged them to increase activity and social contact which the patients felt had improved both their physical health and mood. For example:

*“The telephone conversations for me were helpful. She got me to think about doing things. I’m doing a computer course now and there’s a chance I might be able to help them at [voluntary organisation].”* PT9

*“It has helped me thinking about things I can do… I go in the pool, only in the baby pool but it’s good for my legs and my shoulder… and you know it makes you feel better once you’ve done it, not just my legs, but in yourself, you know…”* PT6

A few patients valued the practical aspects and the techniques learned from the CM:

*“I’ve kept a diary all my working life and by going - a daily diary that is - and by going through it we could highlight various things that tip the balance if you like of the scales of happiness and depression and it was highlighted (depression) and between us we figured out a way of coming through it basically.” PT5*

*“When we moved onto the technical part of it where they are asking specific questions and giving specific ideas, I find these very useful and in fact I’ve continued to do those. The ones I am talking about are where you identify things to do… and make a list.”* PT4

Case management with BA provides older people with tools to help manage their depressive symptoms and to understand that behaviour and mood are closely linked. BA promotes participation in social and physical activity which may enable older people to ‘move on’ from depression and to experience improved wellbeing.

## Discussion

This is the first qualitative study to explore the perspectives of older people, case managers and GPs; all of whom were participants in a trial of collaborative care for older people, the CASPER Plus study. Our findings indicate that depression in older people is commonly hidden, and invitation to participate in a trial can serve to uncover depression in patients and to raise awareness in GPs; interaction with the CM provides older patients with an opportunity to talk outside the primary care consultation, to deal with their low mood and move forwards.

Our findings support the literature suggesting that case management can help to reduce stigma and may improve access to mental health care [23, 26]; certainly being invited to participate in a trial acted as a catalyst for older people to reflect on their feelings and the depression may not have been identified outside the trial setting.

Both GPs and patients may normalise depression and view it as an expected consequence of having one or more chronic health conditions [10, 12]. GPs may be reluctant to address signs and symptoms of the condition, partly due to the lack of treatment options for older depressed adults, and the limited consultation time in which to address the problem. Our results add to the evidence that there is insufficient capacity within existing primary care for psychosocial support of older people with depression [14], and that older people may value a separate space to discuss their problems.

### Strengths and weaknesses

This study explored multiple perspectives on the views and experiences of those receiving and delivering a psychosocial intervention for depression within a CC framework. While our aim was to interview people across a wide demographic range, we were less successful in engaging many from areas of lower socioeconomic status (SES). This, we believe, may be a reflection of social deprivation levels; patient participants constrained by social or financial circumstances were less likely to respond to an invitation to participate in the main trial, and those who did were less likely to agree to take part in an interview after they completed treatment. Similarly, GPs in areas of lower SES were less likely to respond to an invitation to be interviewed. Additionally, black or minority ethnic groups were poorly represented in the population of GP practices participating in the study.

## Conclusions

Depression is commonly hidden and coexists with physical conditions that are prioritised by both patients and GPs. Being invited to participate in a trial about depression seems to facilitate acceptance of symptoms, may reduce stigma and allow older people to disclose their feelings, name the problem, and access care. Older people value an opportunity to talk outside the GP consultation.

Although the results of the CASPER Plus randomised controlled trial are as yet unknown, the findings from this nested qualitative study suggest that a psychosocial intervention delivered by a case manager can provide a valuable resource, which fills a gap in the care of older people with depression. Behavioural Activation (BA) encourages increased activity and social contact which may improve physical health symptoms as well as mood; it can enable older people to ‘move on’ from depression, providing them with the tools to manage their symptoms.

## Abbreviations

BA: Behavioural Activation
CC: Collaborative care
CM: Case manager
PHQ-9: Patient Health Questionnaire 9
RCT: Randomised controlled trial
SES: Socioeconomic status.

## References

[1] McDougall FA, Kvaal K, Matthews FE, Paykel E, Jones PB, Dewey ME, Brayne C (2007). Prevalence of depression in older people in England and Wales: the MRC CFA Study. Psychol Med.

[2] Djernes JK (2006). Prevalence and predictors of depression in populations of elderly: a review. Acta Psychiatr Scand.

[3] Barnett K, Mercer SW, Norbury M, Watt G, Wyke S, Guthrie B (2012). Epidemiology of multimorbidity and implications for health care, research, and medical education: a cross-sectional study. Lancet.

[4] Manthorpe J. Iliffe S, Suicide in older people. Nursing Older People. 2006;17(10):25-9. 10.7748/nop2006.01.17.10.25.c2404 16445224

[5] Dickens C, Katon W, Blakemore A, Khara A, McGowan L, Tomenson B, Jackson J, Walker L, Guthrie E (2012). Does depression predict the use of urgent and unscheduled care by people with long term conditions? A systematic review with meta-analysis. J Psychosom Res.

[6] Heikkinen RL, Kauppinen M (2004). Depressive symptoms in late life: a 10-year follow-up. Arch Gerontol Geriatr.

[7] Burholt V, Scharf T (2014). Poor health and loneliness in later life: the role of depressive symptoms, social resources, and rural environments. J Gerontol B Psychol Sci Soc Sci.

[8] Cacioppo JT, Hughes ME, Waite LJ, Hawkley LC, Thisted RA (2006). Loneliness as a specific risk factor for depressive symptoms: cross-sectional and longitudinal analyses. Psychol Aging.

[9] Burroughs H, Lovell K, Morley M, Baldwin R, Burns A, Chew-Graham C (2006). ‘Justifiable depression’: how primary care professionals and patients view late-life depression? A qualitative study. BMC Fam Pract.

[10] Chew-Graham C, Kovandzic M, Gask L, Burroughs H, Clarke P, Sanderson H, Dowrick C (2012). Why may older people with depression not present to primary care? Messages from secondary analysis of qualitative data. Health Soc Care Community.

[11] Alderson S, Foy R, Glidewell L, House A, Patients’ understanding of depression associated with chronic physical illness: a qualitative study. BMC Fam Pract. 2014;15-17. 10.1186/1471-2296-15-37 PMC3936902 24555886

[12] Coventry P, Hays R, Dickens C, Bundy C, Garrett C, Cherrington A, Chew-Graham C (2011). Talking about depression: a qualitative study of barriers to managing depression in people with long term conditions in primary care. BMC Fam Pract.

[13] Licht-Strunk E, Beekman ATF, de Haan M, van Marwijk HWJ (2009). The prognosis of undetected depression in older general practice patients. A one year follow-up study. J Affect Disord.

[14] Lewis H, Hems D, Bosanquet K, Overend K (2013). Is enough being done to treat depression in the elderly?. Aging Health.

[15] Bristow K, Edwards S, Funnel E, Fisher L, Gask, L, Dowrick, C, et al. Help Seeking and Access to Primary Care for People from “Hard-to-Reach” Groups with Common Mental Health Problems. Int J Fam Med, 2011. 10.1155/2011/490634 PMC3268206 22312546

[16] Ekers D, Richards D, Gilbody S (2008). A meta-analysis of randomized trials of behavioural treatment of depression. Psychol Med.

[17] Samad Z, Brealey S, Gilbody S (2011). The effectiveness of behavioural therapy for the treatment of depression in older adults: a meta-analysis. Int J Geriatr Psychiatry.

[18] Wilson K, Mottram P, Vassilas C (2008). Psychotherapeutic treatments for older depressed people. Cochrane Collaboration.

[19] Richards D, Hill J, Gask L, Lovell K, Chew-Graham C, Bower P, Cape J, Pilling S, Araya R, Kessler D, Bland JM, Green C, Gilbody S, Lewis G, Manning C, Hughes-Morley A, Barkham M (2013). Clinical effectiveness of collaborative care for depression in UK primary care (CADET): cluster randomised controlled trial. BMJ.

[20] Gunn J, Diggens J, Hegarty K, Blashki G (2006). A systematic review of complex system interventions designed to increase recovery from depression in primary care. BMC Health Serv Res.

[21] Katon W, Unützer J (2006). Collaborative care models for depression: time to move from evidence to practice. Arch Intern Med.

[22] Richards DA, Hughes-Morley A, Hayes RA, Araya R, Barkham M, Bland JM, Bower P, Cape J, Chew-Graham CA, Gask L, Gilbody S, Green C, Kessler D, Lewis G, Lovell K, Manning C, Pilling S (2009). Collaborative Depression Trial (CADET): multi-centre randomised controlled trial of collaborative care for depression--study protocol. BMC Health Serv Res.

[23] Coventry P, Hays R, Dickens C, Bundy C, Garrett C, Cherrington A, Chew-Graham C (2015). Integrated primary care for patients with mental and physical multimorbidity: cluster randomised controlled trial of collaborative care for patients with depression comorbid with diabetes or cardiovascular disease. BMJ.

[24] Overend K, Lewis H, Bailey D, Bosanquet K, Chew-Graham C, Ekers D, Gascoyne S, Hems D, Holmes J, Keding A, McMillan D, Meer S, Meredith J, Mitchell N, Nutbrown S, Parrott S, Richards D, Traviss G, Trepel D, Woodhouse R, Gilbody S (2014). CASPER Plus (CollAborative care in Screen-Positive EldeRs with major depressive disorder): study protocol for a randomised controlled trial.. Trials.

[25] Simpson AE, Richards D, Gask L, Hennessy S, Escott D (2008). Patients’ experiences of receiving collaborative care for the treatment of depression in the UK: a qualitative investigation. Ment Health in Fam Med.

[26] Knowles SE, Chew-Graham C, Coupe N, Adeyemi I, Keyworth C, Thampy H, Coventry P (2013). Better together? a naturalistic qualitative study of inter-professional working in collaborative care. Implement Sci.

[27] Knowles SE, Chew-Graham C, Adeyemi I, Coupe N, Coventry PA (2015). Managing depression in people with multimorbidity: a qualitative evaluation of an integrated collaborative care model. BMC Fam Pract.

[28] Glaser BG (1965). The Constant Comparative Method of Qualitative Analysis. Soc Probl.

[29] Ritchie J, Spencer L (1994). Qualitative data analysis for applied policy research.

